# Neonatal Esophageal Perforation: A Comprehensive Review of the Literature

**DOI:** 10.3390/jcm15041603

**Published:** 2026-02-19

**Authors:** Gregorio Serra, Veronica Notarbartolo, Maria Rita Di Pace, Ingrid Anne Mandy Schierz, Valeria Guarneri, Marco Pensabene, Maria Sergio, Mario Giuffrè, Giovanni Corsello

**Affiliations:** 1 Neonatal Intensive Care Unit, Department of Health Promotion, Mother and Child Care, Internal Medicine and Medical Specialties “G. D’Alessandro”, University of Palermo, 90133 Palermo, Italy; gregorio.serra@unipa.it (G.S.); veronica.notarbartolo@policlinico.pa.it (V.N.); valeriaguarneri97@gmail.com (V.G.);; 2 Pediatric Surgery Unit, Department of Health Promotion, Mother and Child Care, Internal Medicine and Medical Specialties “G. D’Alessandro”, University of Palermo, 90133 Palermo, Italy

**Keywords:** esophageal rupture, neonate, preterm, iatrogenic injury, neonatal intensive care, ethics, communication

## Abstract

**Background/Objectives**: Neonatal esophageal perforation (EP) is a rare but potentially life-threatening condition, primarily affecting preterm and very low birth weight infants. Iatrogenic injury—most commonly related to malpositioned naso- or orogastric tubes—represents the leading cause. **Methods**: We conducted a comprehensive review of EP cases diagnosed within the first 28 days of life and reported between 2004 and October 2025 in PubMed and Scopus databases. The analysis focused on clinical presentation, risk factors, diagnostic modalities, management strategies, and outcomes. Only English-language case reports, case series, and observational studies (retrospective, cross-sectional and multicenter analyses) were included. Previously published narrative and systematic reviews were screened for relevant primary studies and contextual comparison, but were not included as primary data sources. Additionally, the emotional impact of iatrogenic complications on neonatal teams was also explored, through a focus on the importance of safety culture, reflective practice, and professional learning. **Results**: A total of 84 neonatal EP cases, were identified across 11 publications. The literature consistently indicates that iatrogenic EP predominantly affects infants born <28 weeks of gestational age and weighing <1000 g. Conservative management is effective in the majority of cases, whereas surgical intervention is reserved for complicated or refractory presentations. Prevention relies on standardized tube insertion techniques and early imaging verification. **Conclusions**: Although rare, neonatal EP demands high clinical vigilance, timely imaging-based diagnosis, and cautious conservative treatment. This review aims to consolidate available evidence while emphasizing the role of preventive strategies, safety culture, and team awareness in neonatal intensive care. By integrating clinical findings with reflections on iatrogenic risk, it seeks to support standardized practices, multidisciplinary learning, and continuous improvement in patient safety.

## 1. Introduction

Esophageal perforation (EP) in neonates is an uncommon but potentially life-threatening complication, posing both diagnostic and therapeutic challenges. Reported incidence rates range from 0.05% to 0.15% in the general neonatal population, but the condition is considerably more prevalent among very low birth weight (VLBW, <1500 g) and extremely low birth weight (ELBW, <1000 g) infants [1,2,3]. Mortality rates have been reported between 21% and 30%, reflecting the inherent fragility of this patient population [2]. Most neonatal EPs are iatrogenic in origin, typically resulting from traumatic or misplaced insertion of naso- or orogastric tubes (NGT/OGT), endotracheal intubation, or forceful pharyngeal suctioning [1,4,5]. The esophageal wall in preterm neonates is extremely delicate, with minimal submucosal support and a lack of serosa, making it particularly susceptible to perforation. Additional risk is conferred by anatomical factors (e.g., narrow esophageal lumen, high cervical angulation), repeated procedural attempts, and the frequent use of mechanical ventilation [6,7]. Despite the clinical significance of neonatal EP, existing reviews provide useful insights but are limited by heterogeneous study populations, inclusion of mixed pediatric age groups, and lack of attention to safety-culture considerations. Available data remain confined to case reports, case series, and a small number of retrospective studies—mostly single-center—and a single cross-sectional study, which collectively hamper the development of standardized diagnostic and therapeutic guidelines.

The present comprehensive review aims to (i) synthesize and critically appraise the available literature on neonatal esophageal perforation published between 2004 and 2025, and (ii) outline current diagnostic and therapeutic approaches.

## 2. Materials and Methods

A comprehensive narrative review was conducted to identify all published cases and case series of neonatal EP between January 2004 and October 2025. Two databases—PubMed and Scopus—were searched using combinations of the following keywords: “esophageal perforation”, “newborn”, “neonate”, “preterm”, “iatrogenic injury”, “nasogastric tube”, “orogastric tube”, and “tube malposition.” The search was limited to articles in English. Reference lists of relevant papers were also screened to identify additional publications. Specifically, secondary searches identified large case series and reports of uncommon anatomical sites that were not captured in the initial database queries. Included studies comprised case reports, case series, observational studies (retrospective and cross-sectional analyses) and a multicenter study, reporting EP in neonates aged ≤28 days, regardless of gestational age or birth weight.

Exclusion criteria were non-peer-reviewed materials, editorials, animal studies, and reports describing esophageal perforation beyond the neonatal period. From each eligible publication, data were extracted on:

- gestational age (GA) and birth weight (BW);
- sex, delivery mode, and maternal risk factors;
- associated neonatal conditions;
- etiology and mechanism of perforation;
- diagnostic modalities;
- management strategy (conservative vs. surgical);
- complications and outcomes.

Data were tabulated and synthesized in order to highlight epidemiological trends, diagnostic evolution, and therapeutic outcomes over time. Due to the heterogeneity of the available reports, no formal meta-analysis was performed.

Furthermore, two previously published narrative reviews were explicitly screened during the study selection process. These studies were considered for context; however, they were not included in the quantitative and qualitative synthesis of our results, as they did not meet the predefined inclusion criteria for primary studies reporting original neonatal cases. Such narrative reviews differ from the present work mainly in methodology and structure. Narrative reviews, indeed, typically do not report explicit inclusion or exclusion criteria, and lack a systematic search strategy. Their selection of studies is based on author expertise and contextual discussion, which may introduce subjective or confirmation bias. In contrast, the present review follows a systematic and structured approach, with a clearly defined, replicable search strategy, predefined inclusion and exclusion criteria, structured data extraction, and critical appraisal of included studies. This methodology reduces the risk of bias and ensures a comprehensive, robust, and clinically applicable synthesis of the available evidence.

## 3. Results

### 3.1. Review of Reported Cases (2004–2025)

Between 2004 and October 2025, a total of 84 neonatal cases of esophageal perforation were identified in the literature, across 11 publications. The findings are summarized in Table 1 and Table 2, and a qualitative analysis is discussed in the following paragraphs of this Results section. Nearly all cases involved preterm ELBW and/or VLBW infants (55 and 21 neonates, i.e., 65% and 25%, respectively); 7 were LBW and only 1 was a term newborn with birth weight > 2500 g (9% and 1%, respectively). All were attributable to iatrogenic injury. Over the past two decades, clinical presentation and outcomes have remained broadly stable, apart from a decline in mortality, as diagnostic and management practices have evolved considerably.

### 3.2. Epidemiology and Risk Factors

The cumulative data confirm that neonatal EP predominantly affects male (53.8%), extremely preterm (GA < 28 weeks) and ELBW (<1000 g) infants (65.5%). All perforations were iatrogenic, primarily due to traumatic or malpositioned NGT/OGT insertion (97.6%), although endotracheal intubation and vigorous suctioning were also reported causes (65.5%) [1,2,3,4,5,6,7]. Maternal factors such as preeclampsia, gestational diabetes, premature rupture of membranes (PROM), and chorioamnionitis were frequent antecedents (38%), likely reflecting the perinatal context of preterm birth; twinning, although reported in only two studies and not quantifiable, was also present among maternal risk factors. Among neonatal comorbidities, respiratory distress syndrome (RDS), patent ductus arteriosus (PDA), and sepsis were consistently reported, all contributing to tissue hypoxia and fragility [1,2,3,4,5,6,7,8,9,10,11,12,13,14,15,16,17]. Collectively, these factors exacerbate esophageal vulnerability and complicate recovery. Prematurity and low birth weight, in particular, represent major risk factors that increase tissue susceptibility to iatrogenic injury and to the adverse effects of postnatal therapies. In addition, barotrauma from non-invasive high positive-pressure ventilation—well recognized in the literature as a contributor to EP—may further aggravate injury risk [18]. Other predisposing conditions associated with ischemic or iatrogenic gastrointestinal perforation are also well documented, including inflammatory or infectious diseases (e.g., necrotizing enterocolitis [NEC], congenital infections/tumors directly involving or indirectly compromising the digestive system), malformations (e.g., Boerhaave syndrome, Bochdalek hernia, tracheoesophageal atresia/fistula, diverticula, and strictures), and hypoxia secondary to asphyxia or congenital heart disease [5,19,20,21,22,23,24,25].

**Table 1:** The findings of selected papers.

Authors	Study Design	Cases (n)	GA (Weeks and Median)	BW (Range and Median, gr)	Sex	Type of Delivery	Maternal Factors	Neonatal Factors	Etiology and Additional Risk Factors	Complications and Comorbidities	Diagnostic Modalities	Management	Outcome
**Emil, 2004** **[8]**	Case report	1	26	900	F	Vaginal	N/S	Preterm ELBW	Intubation OGT	RDS, Gastroesophageal reflux	Surgical exploration, Esophagogram, Chest X-ray	Operative [Thoracotomy]	Survival
**Suryawanshi et al., 2014 [9]**	Case report	1	27	900	M	Cesarean section	N/S	Preterm, ELBW	OGT	Sepsis, RDS	Esophagogram	Conservative	Survival
**Hesketh et al., 2015** **[2]**	Case series	7	24–36 (25^+6^)	450–2315 (600)	M (4) F (3)	N/S	Abruptio placentae Chorioamnionitis	Preterm, LBW ELBW, LBW IUGR	OGT, NGT Intubation	Sepsis, NEC, RDS, PDA PNX, PNM, IVH, PNP, Cardiac arrest	Chest X-ray, Tube contrast study, Esophagoscopy, Esophagogram	Conservative (7)	Survival (4), *Exitus* (3)
**Onwuka et al., 2016 [4]**	Observational retrospective study	25	24–29 (26^+5^)	540–1410 (900)	M (13) F (12)	Cesarean section (21) Vaginal (4)	Twinning	Preterm, ELBW, VLBW, twins	OGT, NGT	Sepsis, Pneumonia, PNX, Respiratory failure, Prolonged ventilation, nosocomial infections, NEC, IVH	Esophagogram	Conservative (25)	Survival (21), *Exitus* (4)
**Yong et al., 2016** **[12]**	Case series	3	23–27 (25)	585–995 (650)	N/S	Cesarean Section (2) Vaginal (1)	History of Multiple Abortions	Preterm ELBW	Intubation NGT	Bacteremia, Cerebral ventriculomegaly PNP, Subependymal cyst, RDS, ROP, IVH, PNX	Chest X-ray	Conservative (3)	Survival (3)
**Lithoxopoulou et al., 2019 [13]**	Case report	1	28	1100	M	Cesarean section	Chorioamnionitis, PROM	Preterm, VLBW	NGT (posterior cervical esophageal injury)	Sepsis, RDS, thrombocytopenia	Esophagogram	Conservative	Survival
**Adel et al., 2023** **[3]**	Observational cross-sectional study	15	27–34 (30)	780–1800 (1100)	M (8) F (7)	Cesarean Section (12) Vaginal (3)	Hypertension, PROM Decollement, GDM Placenta previa Preeclampsia	Preterm, LBW ELBW, IUGR VLBW	Intubation OGT	Pneumonia, Sepsis, Tricuspid Regurgitation, LVH, RDS, PDA, ASD, VSD, PNX	Esophagogram	Conservative (15)	Survival (11) *Exitus* (4)
**Mikołajczak et al., 2023** **[14]**	Multicenter retrospective study	10	23–35 (24^+3^)	430–1100 (640)	M (5) F (5)	Cesarean Section (4) Vaginal (6)	Chorioamnionitis	ELBW, VLBW Preterm, SGA IUGR	OGT or NGT Intubation	BPD, PE, Milky PE, Ileum perforation, PDA, Airl leak [PNX, PNM], Sepsis, Renal failure Fungemia, Pneumonia, Cardiorespiratory decompression, IVH, Peritonitis, MOF, RDS, NEC	Chest-X-Ray	Conservative (8) Surgical (2)	Survival (7) *Exitus* (3)
**Sorensen et al., 2023** **[15]**	Multicenter retrospective study	8	23^+4^–39 (26^+4^)	511–3500 (636)	M (7) F (1)	N/S	N/S	Preterm, LBW ELBW	OGT or NGT Intubation	Acute on Chronic Renal Failure, Septic Shock, Pleural effusion, BPD, IVH Mediastinal abscess, RDS, PNX	Chest X-ray	Conservative (8)	Survival (7) *Exitus* (1)
**Aljadaan et al., 2025 [16]**	Case series	7	22^+5^–27 (24^+4^)	460–840 (576)	M (3) F (4)	N/S	N/S	Preterm, ELBW, IUGR Ambiguous genitalia	Intubation OGT	Periventricular leukomalacia, PDA, NEC, Sepsis, Left atrium and ventricle dilatation, BPD, PNX, Lung collapse, Renal failure, ASD, IVH, RDS, PNM, TPN-related liver disease	Surgical exploration Esophagogram Chest X-ray	Conservative (7)	Still hospitalized (1) Survival (2) *Exitus (4)*
**Eguchi et al., 2025** **[17]**	Observational retrospective study	6	23^+5^–28 ^+6^ (27^+1^)	630–1232 (823)	M (4) F (2)	N/S	N/S	Preterm, ELBW VLBW, Triplet SGA	OGT or NGT Intubation	Mediastinitis, PNX, PDA, RDS, IVH	Laryngoscopy Esophagogram Chest X-ray	Conservative (6)	Survival (6)

Note. ASD = Atrial Septal Defect; BPD = Bronchopulmonary Dysplasia; GDM = Gestational Diabetes Mellitus; HCM = hypertrophic cardiomyopathy; LBW = Low Birth Weight; LVH = Left ventricular hypertrophy; MOF = Multi-organ Failure; NEC = Necrotizing Enterocolitis; N/S = Not specified; PE = Pleural effusion; PNM = Pneumomediastinum; PNP= Pneumoperitoneum; PNX = Pneumothorax; PROM= Premature Rupture of Membranes; RDS = Respiratory Distress Syndrome; SGA = Small for Gestational Age; TPN = Total parenteral nutrition VSD = Ventricular Septal Defect.

**Table 2:** Synthesis of demographics, etiology, clinical presentation, management and outcomes of the reviewed 84 neonatal EP cases.

Variable	n (%)
Gestational age (weeks)	Median: 26 Range: 22^+5^–39
Preterm (<37 weeks)	83 (98.8%)
Term (≥37 weeks)	1 (1.2%)
Birth weight (g)	Median: ~650–700 Range: 430–3500
Birth weight categories	ELBW (<1000 g): 55 (65.5%) VLBW (1000–1499 g): 21 (25.0%) LBW (1500–2499 g): 7 (8.3%) >2500 g: 1 (1.2%)
Sex (available for 78/84 cases)	Male: 42 (53.8%) Female: 36 (46.2%) NR: 6 (7.1%)
Maternal risk factors	Maternal infection/inflammation (chorioamnionitis, PROM, abruptio placentae, GDM, hypertension, preeclampsia): 32 (38%), multiple gestation reported in 2 studies (Onwuka et al.; Eguchi et al.) but not quantifiable
Neonatal risk factors	Prematurity/ELBW/VLBW/LBW: 83 (98.8%)
Presumed etiology	OGT/NGT insertion: 82 (97.6%) Endotracheal intubation: 55 (65.5%)
Clinical presentation	Respiratory distress/RDS: 62 (73.8%) Sepsis: 45 (53.6%) Air leak (PNX/PNM): 30 (35.7%) Mediastinitis/pleural effusion: 8 (9.5%)
Age at diagnosis	NR (not consistently reported)
Diagnostic modalities	Esophagogram: 81 (96.4%) Chest X-ray: 76 (90.5%) Endoscopy/Laryngoscopy: 6 (7.1%)
Management	Conservative: 81 (96.4%) Surgical: 3 (3.6%)
Outcome	Survival 65 (77.4%) Mortality/Exitus 19 (22.6%)

Note. ELBW = Extremely low birth weight; GDM = Gestational Diabetes Mellitus; LBW = Low Birth Weight; N/R = Not reported; NGT = nasogastric tube; OGT = orogastric tube; PNM = Pneumomediastinum; PNX = Pneumothorax; PROM = Premature Rupture of Membranes; RDS = Respiratory Distress Syndrome; VLBW = Very low birth weight.

Anatomically, perforations were most frequently located at the pharyngoesophageal junction or in the distal third of the esophagus, where angulation and compression by adjacent vertebral structures create areas of mechanical vulnerability. The absence of a serosal layer facilitates the spread of inflammation and infection into the mediastinum or pleural cavities, occasionally resulting in mediastinitis, pneumothorax, or sepsis (see Discussion section below) [5,11]. While the pharyngoesophageal junction represents the most common site of perforation, clinicians should remain vigilant for atypical locations. Thoracic esophageal perforations, although rare, have been reported following routine orogastric tube placement or replacement in premature infants [13].

### 3.3. Diagnostic Approaches

**Figure 1:**
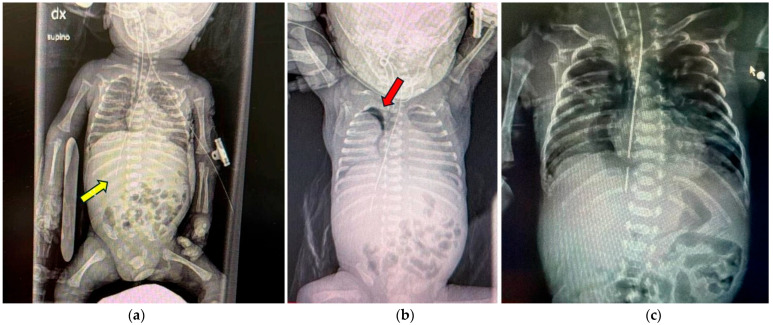
(**a**) Abnormal position of the nasogastric tube, with its tip located in the right hypochondrium at the level of the twelfth rib (yellow arrow). (**b**) In the upright image, a right-sided pneumothorax is visible, with partial lung collapse. The thickness of the air pocket indicated by the red arrow measures 5 mm. (**c**) Pneumothorax collection located in the right paramediastinal region following esophageal perforation in an extremely preterm neonate.

Clinical signs were typically nonspecific, including respiratory distress, cyanosis, abdominal distension, or difficulty advancing a feeding tube. Chest and abdominal radiographs remain the first-line diagnostic tool, revealing misplaced tubes, pneumothorax, or pneumomediastinum (Figure 1a–c).

However, up to one-third of cases initially display normal plain films, underscoring the need for confirmatory imaging [5]. Specifically, plain X-rays may fail to identify subtle mediastinal air or minor perforations, particularly in the cervical region. Over the past decade, point-of-care ultrasonography (POCUS) has emerged as a valuable, radiation-free, and real-time alternative for verifying tube placement and detecting pneumothorax or mediastinal air [26]. Recent reports document sensitivity rates exceeding 90% when performed by trained operators. Contrast esophagography has been found to be reserved for unclear cases or assessment of healing prior to feeding resumption [2].

### 3.4. Management and Outcomes

Conservative treatment—consisting of tube removal, *nil per os*, broad-spectrum antibiotics, parenteral nutrition, and supportive care—was the predominant approach in over 95% of cases [3,15,27]. Surgical treatment was performed in three cases (3.5%) included in the review [8,14]. The surgical approach consisted of direct suturing of the esophageal perforation. No cases of esophagostomy were reported. In patients presenting with abscess or mediastinitis, management was predominantly conservative (including antibiotic therapy, drainage, and nutritional support), while surgery was reserved for complicated and early presentations, prior to the development of diffuse infection. Although the literature suggests that esophageal diversion (esophagostomy) may be required in the presence of mediastinitis or abscess [15], no cases treated with this technique were reported in the included studies. In cases complicated by air leakage (pneumothorax, pneumomediastinum, or pneumoperitoneum), management consisted of thoracic or abdominal drainage combined with conservative treatment, without the need for major surgical intervention [3,4]. Endoscopic treatment, including vacuum-assisted therapy, was not reported as a management option in the neonatal patients described; however, the literature documents its use in selected neonatal and pediatric cases (persistent leakage or anastomotic dehiscence not responsive to conservative management, first-line approach in the presence of contained perforations without signs of sepsis or extensive contamination, rescue therapy following failure of surgical suturing) [28]. The median duration of fasting ranged between 10 and 14 days, with reintroduction of enteral feeding guided by repeat imaging [5,8]. The overall survival rate exceeded 75%, with most fatalities attributable to extreme prematurity and comorbid sepsis. Temporal analysis revealed a decline in mortality from ~35% before 2010 to <20% after 2018, reflecting the increasing use of early imaging (especially US), standardization of feeding tube protocols, and emphasis on operator training. Complications most frequently reported include pneumothorax, pneumomediastinum, empyema, sepsis, and mediastinitis. While the direct mortality attributable to EP is relatively low, the overall prognosis is significantly influenced by gestational age, comorbidities, and infection severity [1,2,3,4,10,11]. Infants with extreme prematurity (<26 weeks) or birth weight <700 g have been detected to have poorer outcomes, largely due to multisystem immaturity rather than the perforation itself.

## 4. Discussion

Neonatal EP remains a rare but critical event, particularly among extremely preterm infants, whose tissue immaturity and fragility predispose them to procedural trauma. The pathophysiological substrate involves the combination of immature connective tissue, lack of a serosal layer, and poor vascularization of the neonatal esophagus, rendering it susceptible even to minimal mechanical stress. The latter, indeed, when associated with routine procedures, such as scheduled tube replacement every 3–5 days, may precipitate sudden esophageal perforation even after previously uncomplicated insertions, as demonstrated in reported neonatal cases [13]. The delicate balance between required intensive interventions—such as airway management and enteral nutrition—and the risk of iatrogenic harm constitutes a recurring dilemma in neonatal intensive care [1,2,3]. The present review explicitly contrasts with prior studies in several key aspects. First, it encompasses a broader spectrum of study designs, including case reports, case series, retrospective observational studies, an observational cross-sectional study, and two multicenter retrospective studies, thereby providing a more comprehensive overview of the literature. Second, it focuses specifically on neonatal patients, whereas previous analyses often included mixed pediatric populations. Third, the time frame of the included papers has been updated to capture the most recent evidence. Finally, the review incorporates considerations related to clinical safety and risk management, highlighting the role of iatrogenic factors and strategies to prevent complications. Indeed, the available evidence emphasizes that iatrogenic injury continues to account for the vast majority of cases. This observation further underscores the necessity of procedural standardization and continuous professional training, particularly for less experienced healthcare providers working in high-stress NICU environments. Traditional radiographic confirmation of tube placement has long been considered the diagnostic cornerstone, but it carries limitations. Furthermore, contrast-enhanced imaging remains indispensable for complex cases, not only confirming the diagnosis but also guiding the decision to resume enteral feeding. However, its use must be judicious, balancing diagnostic yield against the risk of aspiration or further mucosal injury. The growing integration of multimodal imaging aligns with the trend toward minimally invasive diagnostics in neonatology, promoting earlier detection and safer management [26].

The predominant treatment paradigm for neonatal EP is conservative management, which has demonstrated high success rates when initiated promptly. The safety and effectiveness of nonoperative management are further supported by Onwuka et al., who reported a 100% survival rate in a cohort of 25 neonates managed conservatively [4]. Supportive endotracheal ventilation and fluid balance are essential components of care. In contrast, surgical intervention should be reserved for cases complicated by abscess, mediastinitis, or uncontrolled leakage. Surgical repair in this population is technically challenging and associated with higher mortality; therefore, it is justified only when conservative therapy fails or life-threatening complications occur [3,4,5,11]. Recent literature indicates that the duration of fasting and timing of feeding resumption can be individualized based on imaging findings [5,8]. Early follow-up with contrast studies or US to confirm healing is now considered best practice. Importantly, interdisciplinary coordination among neonatologists, pediatric surgeons, and radiologists plays a pivotal role in optimizing outcomes. Notably, several recent series demonstrate a progressive decline in mortality since 2015. While earlier reports have credited this improvement to the increasing use of early imaging, infection control, and nutritional support [1,7], it is likely that advances in general aspects of neonatal intensive care—including respiratory management, hemodynamic support, analgesia and sedation, the use of less invasive devices and life-support systems, and the implementation of less traumatic procedural tools—have played a more critical role in enhancing survival.

Considering its predominantly iatrogenic etiology, prevention remains the most effective strategy against neonatal esophageal perforation (EP). Recommended measures include:

1. Gentle NGT/OGT insertion techniques, using minimal force and adequate lubrication;
2. Extending intervals between routine tube replacements, which may reduce cumulative mechanical stress on the fragile neonatal esophageal wall;
3. Accurate tube length estimation based on measurement methods (e.g., NEMU [Nose–Ear–Mid–Xiphoid–Umbilicus] or NEX [Nose–Earlobe–Xiphoid]);
4. Routine post-insertion verification via radiography or ultrasonography before initiating feeding;
5. Minimizing repeated insertion attempts, particularly in unstable or extremely preterm infants;
6. Simulation-based procedural training for NICU staff to reinforce safe handling of fragile neonates [18,29].

While NGT/OGT insertion has traditionally been described as the predominant trigger event, endotracheal intubation is equally important and should be given similar consideration. As both procedures are typically performed within a similar timeframe on the first day of life, it is often difficult to ascribe the perforation to one procedure over the other. Prevention strategies should therefore factor in both procedures. Moreover, the use of less traumatic devices for intubation, such as plastic blades (e.g., Parker Neonatal Video Laryngoscope), may further reduce the risk of iatrogenic injury.

Despite these recommendations [30], the literature still lacks standardized diagnostic algorithms and unified management guidelines for neonatal EP. Previous reviews on neonatal esophageal perforation have provided valuable insights but also exhibit notable limitations. Many included only a small number of cases, restricting the generalizability of their conclusions. Several reviews combined neonatal and broader pediatric populations, limiting the ability to draw neonatal-specific inferences. Outcome reporting was often inconsistent, with incomplete data on management strategies, procedural details, and clinical follow-up. In particular, incomplete follow-up data hinder a full understanding of potential late sequelae, such as esophageal strictures, dysmotility, or persistent feeding difficulties. Taken together, the limited number of reported cases, heterogeneity in reporting, and underrepresentation of long-term outcomes have, to date, constrained the generalizability of the findings.

### Safety Culture, Reflective Practice, and Professional Learning

In addition to technical precautions, fostering a strong safety culture—one in which adverse events are discussed transparently and reframed as learning opportunities—has been shown to reduce procedural complications. Institutional strategies such as standardized checklists, peer review, and non-punitive error-reporting systems promote accountability and continuous improvement. Every case of neonatal EP should, then, also prompt reflective practice—a structured process through which healthcare professionals analyze the event, identify modifiable factors, and translate lessons into improved clinical protocols [29,30]. The emotional impact of iatrogenic complications on neonatal teams can be substantial, often leading to self-doubt or moral distress. Implementing peer-support programs and clinical supervision frameworks helps mitigate these effects, enabling clinicians to regain confidence and professional balance. Furthermore, incorporating clinical simulation, morbidity-and-mortality conferences, and ethical reflection groups into NICU routines can transform individual errors into institutional learning, ultimately strengthening both patient safety and team resilience [19,29,30,31,32].

## 5. Conclusions

Esophageal perforation in ELBW preterm infants remains a rare but potentially life-threatening complication, often associated with iatrogenic interventions such as NGT/OGT placement. The management of neonatal EP has evolved from high surgical dependency to a conservative, multidisciplinary, and ethically aware approach [33,34,35,36,37,38,39,40]. Early recognition (imaging-guided diagnosis, especially bedside US), prompt withdrawal of the offending device, and individualized supportive care have significantly improved survival outcomes. However, the overall prognosis in such patients is strongly influenced by prematurity-related vulnerabilities, including respiratory immaturity, susceptibility to infections, and multiorgan fragility. Therefore, prevention remains the cornerstone of care. Continued reporting of such cases enriches the body of evidence guiding best practices in neonatal intensive care. It also underscores the relevance of vigilance, standardized NICU protocols, including early detection, multidisciplinary coordination and meticulous procedural execution, as well as ongoing staff training to prevent complications.

## Data Availability

The original data presented in the study are openly online available at https://pubmed.ncbi.nlm.nih.gov.

## Abbreviations

BW	birth weight
ELBW	extremely low birth weight
E.P	Esophageal perforation
GA	gestational age
NEC	necrotizing enterocolitis
NGT/OGT	naso- or orogastric tubes
PDA	patent ductus arteriosus
POCUS	point-of-care ultrasonography
PROM	premature rupture of membranes
RDS	espiratory distress syndrome
VLBW	very low birth weight

## References

[1] Elgendy M.M., Othman H., Aly H. (2021). Esophageal Perforation in Very Low Birth Weight Infants. Eur. J. Pediatr..

[2] Hesketh A.J., Behr C.A., Soffer S.Z., Hong A.R., Glick R.D. (2015). Neonatal esophageal perforation: Nonoperative management. J. Surg. Res..

[3] Adel M.G., Sabagh V.G., Sadeghimoghadam P., Albazal M. (2023). The Outcome of Esophageal Perforation in Neonates and Its Risk Factors: A 10-Year Study. Pediatr. Surg. Int..

[4] Onwuka E.A., Saadai P., Boomer L.A., Nwomeh B.C. (2016). Nonoperative Management of Esophageal Perforations in the Newborn. J. Surg. Res..

[5] Rentea R.M., St Peter S.D. (2017). Neonatal and Pediatric Esophageal Perforation. Semin. Pediatr. Surg..

[6] Seefelder C., Elango S., Rosbe K.W., Jennings R.W. (2001). Oesophageal Perforation Presenting as Oesophageal Atresia in a Premature Neonate Following Difficult Intubation. Paediatr. Anaesth..

[7] Hermans K.E., Witvliet M.J., van den Hoogen A., de Bijl-Marcus K.A. (2025). A Case Series of Iatrogenic Gastrointestinal Perforations in Premature Infants Below 30 Weeks’ Gestation. J. Pediatr. Surg..

[8] Emil S.G.S. (2004). Neonatal esophageal perforation. J. Pediatr. Surg..

[9] Suryawanshi P., Dahat A., Nagpal R., Malshe N., Kalrao V. (2014). A rare case of accidental esophageal perforation in an extremely low birth weight neonate. J. Clin. Diagn. Res..

[10] Hodgson K., Togo A., Moore A.M., Moody A., King S.K., Zani A. (2018). Neonatal Oesophageal Perforation: The Role for Non-Operative Management. J. Paediatr. Child Health.

[11] Gander J.W., Berdon W.E., Cowles R.A. (2009). Iatrogenic esophageal perforation in children. Pediatr. Surg. Int..

[12] Yong S.B., Ma J.S., Chen F.S., Chung M.Y., Yang K.D. (2016). Nasogastric Tube Placement and Esophageal Perforation in Extremely Low Birth Weight Infants. Pediatr. Neonatol..

[13] Lithoxopoulou M., Gkampeta A., Babatseva E., Simeoforidou E., Chatzitoliou E., Georgiadou P., Anastasiadis K., Kaselas C., Kepertis C., Spyridakis I. (2019). Unusual site for iatrogenic esophageal perforation in a premature neonate. J. Pediatr. Neonat. Individual. Med..

[14] Mikołajczak A., Kufel K., Żytyńska-Daniluk J., Rutkowska M., Bokiniec R. (2023). Iatrogenic Esophageal Perforation in Premature Infants: A Multicenter Retrospective Study from Poland. Children.

[15] Sorensen E., Yu C., Chuang S.L., Midrio P., Martinez L., Nash M., Jester I., Saxena A.K. (2023). Iatrogenic Neonatal Esophageal Perforation: A European Multicentre Review on Management and Outcomes. Children.

[16] Aljadaan S.A., Alharbi N.S., Alnamshan M.K., Alaqeel S.M., Abaas A.O. (2025). Iatrogenic esophageal perforation in extremely premature babies with low birth weight: A case series at a single tertiary-care center. Cureus.

[17] Eguchi S., Hisaeda Y., Ukawa T., Koto M., Hosokawa M., Tsurisawa C., Takeda T., Amagata S., Nakao A. (2025). Clinical Features of iatrogenic Pharyngo-esophageal perforation in very low birth weight infants. Pediatr. Neonatol..

[18] Sapin E., Gumpert L., Bonnard A., Carricaburu E., Sava E., Contencin P., Helardot P. (2000). Iatrogenic pharyngoesophageal perforation in premature infants. Eur. J. Pediatr. Surg..

[19] Kan S.Y., Ngeow A.J.H., Tan M.G., Jacobsen A.S., Sanamandra S.K., Poon W.B. (2024). Esophageal Perforation in VLBW Infants in a Singapore Tertiary Hospital: A Case-Control Study. Ann. Pediatr. Res..

[20] Savarino G., Carta M., Cimador M., Corsello A., Giuffrè M., Schierz I.A.M., Serra G., Corsello G. (2021). Necrotizing enterocolitis in the preterm: Newborns medical and nutritional Management in a Single-Center Study. Ital. J. Pediatr..

[21] Giuffrè M., Lo Verso C., Serra G., Moceri G., Cimador M., Corsello G. (2016). Study Group of Neonatal Infectious Diseases Affiliated to the Italian Society of Neonatology Portal Vein Thrombosis in a Preterm Newborn with Mutation of the MTHFR and PAI-1 Genes and Sepsis by *Candida parapsilosis*. Am. J. Perinatol..

[22] Schierz I.A.M., Giuffrè G., Cimador M., D’Alessandro M.M., Serra G., Favata F., Antona V., Piro E., Corsello G. (2022). Hypertrophic pyloric stenosis masked by kidney failure in a male infant with a contiguous gene deletion syndrome at Xp22.31 involving the steroid sulfatase gene: Case report. Ital. J. Pediatr..

[23] Serra G., Antona V., Giuffré M., Li Pomi F., Lo Scalzo L., Piro E., Schierz I.A.M., Corsello G. (2021). Novel missense mutation of the TP63 gene in a newborn with Hay-Wells/Ankyloblepharon-Ectodermal Defects-Cleft Lip/Palate (AEC) syndrome: Clinical report and follow-up. Ital. J. Pediatr..

[24] Grasso F., Baldanza F., Pernicone S., Pensabene M., Sergio M., Di Pace M.R. (2025). The Role of Endoscopy in the Postoperative Management of Patients Treated for Esophageal Atresia: 20 Years of Experience. Diagnostics.

[25] Serra G., Cimador M., Giuffrè M., Insinga V., Montante C., Pensabene M., Piro E., Salerno S., Schierz I.A.M., Corsello G. (2023). Report and follow-up on two new patients with congenital mesoblastic nephroma. Ital. J. Pediatr..

[26] Atalay Y.O., Polat A.V., Ozkan E.O., Tomak L., Aygun C., Tobias J.D. (2019). Bedside ultrasonography for the confirmation of gastric tube placement in the neonate. Saudi J. Anaesth..

[27] Thanhaeuser M., Lindtner-Kreindler C., Berger A., Haiden N. (2019). Conservative Treatment of Iatrogenic Perforations Caused by Gastric Tubes in Extremely Low Birth Weight Infants. Early Hum. Dev..

[28] Kaczmarek D.J., Heling D.J., Strassburg C.P., Katzer D., Düker G., Strohm J., Müller A., Heydweiller A., Weismüller T.J. (2022). Management of esophageal perforations in infants by endoscopic vacuum therapy: A single center case series. BMC Gastroenterol..

[29] Panagos P.G., Pearlman S.A. (2017). Creating a Highly Reliable Neonatal Intensive Care Unit Through Safer Systems of Care. Clin. Perinatol..

[30] Caeymaex L., Astruc D., Biran V., Marcus L., Flamein F., Le Bouedec S., Guillois B., Remichi R., Harbi F., Durrmeyer X. (2022). An Educational Programme in Neonatal Intensive Care Units (SEPREVEN): A Stepped-Wedge, Cluster-Randomised Controlled Trial. Lancet.

[31] Hybinette K., Pukk Härenstam K., Ekstedt M. (2021). A First-line management team’s strategies for sustaining resilience in a specialised intensive care unit-a qualitative observational study. BMJ Open.

[32] Bondurant P.G., Nielsen-Farrell J., Armstrong L. (2015). The Journey to High Reliability in the NICU. J. Perinat. Neonatal Nurs..

[33] Quandt D., Schraner T., Ulrich Bucher H., Arlettaz Mieth R. (2009). Malposition of feeding tubes in neonates: Is it an issue?. J. Pediatr. Gastroenterol. Nutr..

[34] Ottosen M.J., Engebretson J., Etchegaray J., Arnold C., Thomas E.J. (2019). An Ethnography of Parents’ Perceptions of Patient Safety in the Neonatal Intensive Care Unit. Adv. Neonatal Care.

[35] Arimitsu T., Hatayama K., Gaughwin K., Kusuda S. (2025). Ethical Considerations Regarding the Treatment of Extremely Preterm Infants at the Limit of Viability: A Comprehensive Review. Eur. J. Pediatr..

[36] Janvier A., Barrington K., Farlow B. (2020). Multidisciplinary ethics support in neonatal intensive care. J. Med. Ethics.

[37] Albersheim S. (2020). The extremely preterm infant: Ethical considerations in life and death decision making. Front. Pediatr..

[38] Boss R.D., Hutton N., Sulpar L.J., West A.M., Donohue P.K. (2017). Family-centered communication in neonatal intensive care. J. Perinatol..

[39] Wilkinson D., Pike K., Savulescu J. (2019). Decision-making in extremely preterm infants. Arch. Dis. Child. Fetal Neonatal Ed..

[40] Bluebond-Langner M., Beecham E., Candy B., Langner R., Jones L. (2018). Shared decision-making in the NICU. Pediatrics.

